# The role of allochrony in influencing interspecific differences in foraging distribution during the non-breeding season between two congeneric crested penguin species

**DOI:** 10.1371/journal.pone.0262901

**Published:** 2022-02-09

**Authors:** Cara-Paige Green, Norman Ratcliffe, Thomas Mattern, David Thompson, Mary-Anne Lea, Simon Wotherspoon, Pablo Garcia Borboroglu, Ursula Ellenberg, Kyle W. Morrison, Klemens Pütz, Paul M. Sagar, Philip J. Seddon, Leigh G. Torres, Mark A. Hindell

**Affiliations:** 1 Institute for Marine and Antarctic Studies, University of Tasmania, Hobart, Tasmania, Australia; 2 British Antarctic Survey, Cambridge, United Kingdom; 3 New Zealand Penguin Initiative, Dunedin, New Zealand; 4 Department of Zoology, University of Otago, Dunedin, New Zealand; 5 Global Penguin Society, Puerto Madryn, Chubut, Argentina; 6 National Institute of Water and Atmospheric Research Ltd., Hataitai, Wellington, New Zealand; 7 Australian Centre for Excellence in Antarctic Science, University of Tasmania, Hobart, Tasmania, Australia; 8 Australian Antarctic Division, Department of Agriculture, Water and the Environment, Kingston, Tasmania, Australia; 9 Centro para el Estudio de Sistemas Marinos (CESIMAR–CONICET), Puerto Madryn, Chubut, Argentina; 10 Department of Ecology, Environment and Evolution, La Trobe University, Melbourne, Australia; 11 Antarctic Research Trust, Bremervörde, Germany; 12 National Institute of Water and Atmospheric Research Ltd., Christchurch, New Zealand; 13 Department of Fisheries and Wildlife, Marine Mammal Institute, Oregon State University, Newport, Oregon, United States of America; Phillip Island Nature Parks, AUSTRALIA

## Abstract

Mechanisms promoting coexistence between closely related species are fundamental for maintaining species diversity. Mechanisms of niche differentiation include allochrony which offsets the peak timing of resource utilisation between species. Many studies focus on spatial and temporal niche partitioning during the breeding season, few have investigated the role allochrony plays in influencing interspecific segregation of foraging distribution and ecology between congeneric species during the non-breeding season. We investigated the non-breeding migrations of Snares (*Eudyptes robustus*) and Fiordland penguins (*Eudyptes pachyrhynchus*), closely related species breeding between 100–350 km apart whose migration phenology differs by two months. Using light geolocation tracking, we examined the degree of overlap given the observed allochrony and a hypothetical scenario where the species commence migration simultaneously. We found that Fiordland penguins migrated to the Sub-Antarctic Frontal Zone and Polar Frontal Zone in the austral autumn whereas Snares penguins disperse westwards staying north of the Sub-Tropical Front in the austral winter. Our results suggest that allochrony is likely to be at the root of segregation because the relative profitability of the different water masses that the penguins forage in changes seasonally which results in the two species utilising different areas over their core non-breeding periods. Furthermore, allochrony reduces relatively higher levels of spatiotemporal overlap during the departure and arrival periods, when the close proximity of the two species’ colonies would cause the birds to congregate in similar areas, resulting in high interspecific competition just before the breeding season. Available evidence from other studies suggests that the shift in phenology between these species has arisen from adaptive radiation and phenological matching to the seasonality of local resource availability during the breeding season and reduced competitive overlap over the non-breeding season is likely to be an incidental outcome.

## Introduction

A key problem in ecological theory is understanding how species diversity arises and how it is maintained over time [1, 2]. The mechanisms that promote coexistence between closely related species are central to understanding influences of interspecific interactions, community organisation and the evolutionary processes [3, 4] which contribute to forming the global scale patterns of species richness and distribution. However, the mechanisms underpinning coexistence between individuals and species within an ecological community are still poorly understood [5–7].

Morphologically similar species that share an area provide a useful model for understanding mechanisms that allow coexistence. According to the theory of phylogenetic niche conservatism, closely related species are expected to be similar in terms of their ecological niches [8] but when they exploit a common, limiting resource, interspecific competition will arise that prevents their stable coexistence [9]. To reduce this theoretical competition, niche theory implies that one of the species will need to switch the resource that is utilised (dietary partitioning) or exploit the same resource along different temporal or spatial axes (conditional partitioning) [10–12]. However, competition is not the only mechanism that results in niche differentiation. Niche differentiation may have evolved between closely related species that have adapted to different environmental conditions during geographically isolated speciation and maintain those differences after secondary contact [13].

Spatial segregation involves using similar resources at similar times in different locations. Temporal segregation includes allochrony, which offsets the peak timing of resource utilisation between sympatric species [14] that feed on similar prey, allowing them to utilise the same areas but at different times. Theoretically, for coexistence to occur, competing species need to find a balance between matching their biological needs with the timing of the environmental drivers responsible for promoting the abundance of a shared resource and the amount of interspecific competition for that resource [15–17]. While there have been many studies which have focused on both spatial and temporal partitioning in the reduction of niche overlap during the breeding season, few have investigated the role allochrony plays in influencing interspecific differences in the non-breeding foraging distributions between ecologically similar species [14, 18, 19].

Marine predators, such as seabirds, face different constraints during the breeding and non-breeding seasons. Most seabirds breed in colonies and foraging is confined to a restricted radius around this central place owing to the need to return to the nest to care for offspring, which may lead to high intra- and interspecific competition between individuals and species [20, 21]. Once breeding is over and central place constraints are relaxed one would expect sympatric species with similar resource requirements to disperse into the wider environment at low densities and intermix as resource limitation and competition will be reduced. Nonetheless, evidence suggests that spatial segregation can continue during this period [22, 23], which may be due to competition or differences in habitat preference that evolved during periods when the species were in isolation.

Penguins of the genus *Eudyptes* occupy broadly similar ecological niches, feeding primarily on swarming crustaceans and myctophid fish [24] within the top 13–60 m of the water column [25]. Several eudyptids breed in sympatry and exhibit allochrony such as Macaroni penguins (*Eudyptes chrysolophus*) and Eastern rockhopper penguins (*Eudyptes chrysocome filholi*) at Marion Island [26], Royal penguins (*Eudyptes schlegeli)* and Eastern rockhopper penguins at Macquarie Island [27], and Erect-crested penguins and Eastern Rockhopper penguins on Antipodes Island [28]. Additionally, they show strong stage-specific central place constraints [29–31]. *Eudyptes* penguins are therefore good models to study niche partitioning and coexistence in relation to allochrony.

In this study we investigated two *Eudyptes* penguins endemic to the New Zealand region, Snares penguins (*Eudyptes robustus*) and Fiordland penguins/tawaki (*Eudyptes pachyrhynchus*). These two species have diverged recently and are still morphologically very similar [32] and breed within 100–350 km of each other. Consequently, while not strictly sympatric, their movements during the non-breeding season are sufficiently large relative to the geographic separation of their colonies to allow substantial spatial overlap in non-breeding foraging areas.

Snares and Fiordland penguins both have stable population trends and are similar in many life history traits but show a marked difference in the timing of their annual cycles. As is typical of most *Eudyptes* penguin species, the post-moult non-breeding period of Snares penguins occurs during the austral winter, whereas that of Fiordland penguins starts two months earlier, with the majority of their post-moult non-breeding period taking place in the austral autumn [33, 34]. Seabirds have evolved different strategies to cope with the seasonal variation in productivity in polar regions [14, 22] which may play a role in the habitat selection, spatial distribution and hence niche partitioning between Snares and Fiordland penguins.

We studied the post-moult migrations of Snares and Fiordland penguins to determine the relative importance of spatial and temporal niche partitioning of their non-breeding distribution. Our aims were to: (1) quantify spatial and temporal overlap during the post-moult migrations under the observed level of allochrony and (2) examine how segregation patterns change in a scenario where both species initiate their non-breeding migrations simultaneously. We use these comparisons to infer the relative importance of differences in habitat selection and allochrony in separating the distributions of the non-breeding seasons of the two species.

## Methods

### Field work and geolocation

Tagging work at Snares Island was conducted under permit issued by the New Zealand Department of Conservation (Wildlife Act Authority 35682-FAU) and was approved by the animal ethics committee at the National Institute of Water and Atmospheric Research in New Zealand. Tagging work of Fiordland penguins was conducted under permit issued by the New Zealand Department of Conservation (permit number RES-38882) and was approved by the animal ethics committee at the University of Otago in New Zealand (AEC-04/14). The duration of handling times of all penguins lasted less than 10 minutes and all field team members involved were experienced and minimised stress to the animals during tag deployment and retrieval.

The differences in the phenology of the two species are consistent through time and an accepted aspect of their natural history [33, 34]. Migration periods are consistently offset between the species. Once *Eudyptes* penguins finish their breeding season, they go on a pre-moult trip which lasts between four to eight weeks. They return ashore to perform what is known as a catastrophic moult, when they moult all their feathers at once. The moult takes places over three to four weeks during which the penguins have no waterproofing and cannot go to sea. Once the birds have moulted, they embark on their post-moult migration and will not return ashore again until the following breeding season.

**Fig 1:**
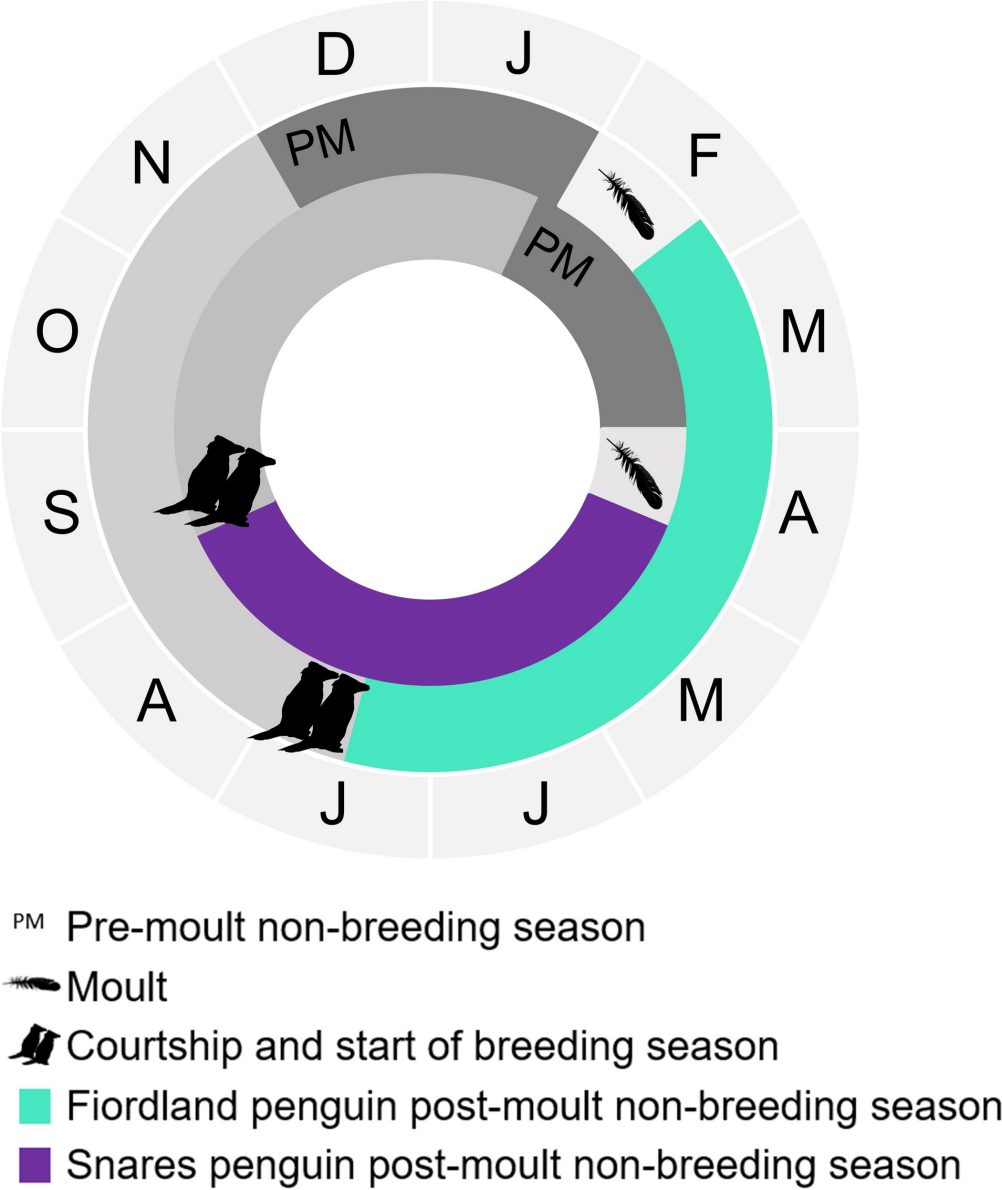
Phenology of Snares penguins and Fiordland penguins, highlighting the post-moult non-breeding seasons. As is typical of *Eudyptes* species, Snares penguins (purple) begin their non-breeding season at the end of April while Fiordland penguins (turquoise) begin their non-breeding season around 8 weeks before, at the end of February.

Around half of the pre-moult trip of Snares penguins overlaps with the first part of the post-moult migration of Fiordland penguins. We could therefore expect some overlap between them during these periods as both species are likely to be near their colonies. Unfortunately, we do not have pre-moult data for Snares penguins and so could not include this in our study. The post-moult migrations are the focus of this study and will be referred as the non-breeding season (Fig 1).

Fieldwork on Snares penguins was conducted on North-East Island (48.016˚ S, 166.533˚ E) in April 2013 and on Fiordland penguins at Jackson Head (45.863˚ S, 170.553˚ E), Harrison Cove (44.674˚ S, 167.923˚ E) and Codfish Island (45.862˚ S, 170.552˚ E) in September 2017. Devices were deployed on Snares penguins at the end of the moult in April and retrieved when the penguins returned to breed the following September, thus only spanning the non-breeding period. For Fiordland penguins, devices were deployed at the beginning of the breeding season in August and were left on the birds for a year. While tags were deployed at the end of 2017, the data spanning the non-breeding season the following year (2018) was used.

Sampling occurred in different years. If changes in oceanography over time alter the distributions of the species among years, it would compromise our comparisons of spatio-temporal overlap. However, we expect that the changes in distribution within species across years will be smaller than the differences between species within years. This is because we know that other *Eudyptes* penguins from the same colonies have consistent migration routes and foraging habitats during their non-breeding season across multiple years [35–37]. Further, the non-breeding distribution of Fiordland penguins are consist across the pre-moult and post-moult dispersals across multiple years [38, 39]. Finally, there was little difference in the locations of the oceanic fronts between the two years which suggests that the major oceanographic features such as frontal zones and eddies which might influence penguin distribution did not differ greatly between the two years (S1 Fig).

Geolocation devices were deployed on 52 Fiordland penguins and 44 Snares penguins (MK3005 Biotrack Ltd., Wareham, UK, 16 × 14 × 6 mm and 2.5 g). These devices recorded light level, time, sea temperature and activity by means of salt-water immersion (wet *vs* dry state: *e*.*g*. Mattern et al. [40]). Birds were captured at their nests and sexed using bill length and depth measurements [33, 34]. For both species, tags were attached to their legs using encased cable ties [41]. The devices deployed on Snares penguins failed to record temperature accurately, so SST adjustments were not possible for their tracks.

The tag data were processed using the R packages *SGAT* and *BAStag* [42, 43]. We used a threshold method that defines twilights as times when light levels have passed a certain threshold. The solar elevation (zenith of the sun at the colony) relates this threshold to the sun’s angle relative to the earth at the horizon for both sunrise and sunset, which then infers geographic positions from algorithms. Initially, the times of the twilights are recognised in *BAStag*, which automatically detects when the threshold is crossed and allows the user to adjust times to account for obvious shading events. Thereafter, location estimates are provided based on times of twilights inferred from the light data and three priors. The first prior is a gamma distribution of penguin swimming speeds to restrict the distances between consecutive locations. The second prior was a beta distribution of errors of the estimated times of twilight. The third prior was a Gaussian distribution of water temperatures recorded by the tag which was used to constrain potential locations by comparison to remotely sensed sea surface temperature (SST) obtained from The National Oceanic and Atmospheric Administration (https://psl.noaa.gov). This is useful for constraining latitude during the equinoxes, when daylength is uniform across the globe. This third prior was not included in the analysis of the Snares penguin data because a fault in the temperature chip in their batch of tags meant no SST data were collected. This had little impact on the quality of the tracks as the migration period of the species does not overlap with the solar equinoxes. Latitude is calculated from day length and longitude is calculated from the time of midday and midnight relative to Coordinated Universal Time. So, the latitude estimation of positions for the Snares tracks from light alone was sufficiently accurate without SST priors.

### Movement parameters

We calculated two sets of location outputs: one corresponding to the mean location at the time instant of each twilight (S2 Fig) and another encompassing all potential locations for the animal during the inter-twilight periods (S3 Fig), given uncertainties in its movement rate and trajectory between each known twilight location. Geolocation estimates have a low precision (50–80 km for non-flying marine animals) [44], so it is important to retain the uncertainty in distribution and overlap metrics. The posterior distribution provides the full range of location estimates between each sunrise and sunset from 4 Bayesian Markov Monte Carlo chains of 3,000 iterations each–a total of 12,000 potential location estimates between each twilight (representing an approximate 12-hour period). This second output was used to calculate spatial distribution and utilisation distributions as it best represents the relative importance in a unit of space with uncertainty; the former output was used to calculate trip metrics.

We derived a two-dimensional Kernel Density Estimate, using the “kde” method in *SGAT*, using all intermediate location estimates across all individuals and chains and then binned these full posterior distribution estimates into percentiles to represent the core and the peripheral areas of use of the animals. To produce the distribution estimate that represented the 50% utilisation distribution (UD), we split the full posterior distribution estimates by the 50^th^ percentile. We then enclosed this top 50^th^ percentile of data with a polygon to produce an isopleth that encompassed the area where 50% of the distribution estimates were located. The same was done for the 90th percentile to produce the 90% UD. In this way, we preserved all location estimates per location for each point in between the fixed twilights. For ease of reference, these will be referred to as the 50% UD and the 90% UD. The data for the mainland and Codfish Island colonies of the Fiordland penguins were pooled for the purposes of this study as the UDs were sufficiently similar (S4 Fig) for inter-species comparison.

The proportion of locations found within the distinct inter-Oceanic frontal zones was calculated using the full range of posterior location outputs for the full migration and dynamic sea surface height extracted from the Copernicus Marine Environment Monitoring Service (marine.copernicus.eu). To determine the usage of the seas in terms of different frontal zones, we calculated the proportion of locations within each of the frontal zones as a percentage of the full posterior distribution locations within the 90% UD. The locations of the fronts were calculated by using the southern extents of the fronts defined by sea surface height contours following Venables et al. [45]. The southern extent of the Sub-Tropical Front [46] and the Sub-Antarctic Front were defined as 0.5 dyn cm and 0.128 dyn cm, respectively, and the Polar Front and Southern Antarctic Circumpolar Current Front as -0.634 dyn cm and -1.09 dyn cm, respectively [45]. This approach allows the annual and seasonal location of ocean fronts to be determined [47]. To calculate the front locations for the periods over which the birds were tracked, we used one mean dynamic height value for each front for February—June 2018 for Fiordland penguins and April—September 2013 for Snares. Finally, bathymetry used in all maps was extracted from the GEBCO_08 Grid (http://www.gebco.net).

We used the mean coordinates for each twilight (rather than the full posterior of intermediate locations) for calculation of trip metrics. As penguins do not come ashore over the duration of their non-breeding season, we considered the birds to have departed the colony once the activity data registered 24 hours of being fully immersed (used to calculate mean departure date) and similarly, they were considered to have returned to their breeding colony once the activity data registered 24 hrs of being dry (use to calculate mean return date). The furthest distance from the colony and the cumulative distance travelled during the non-breeding season were calculated using the great circle route. We related trip metrics to species and sex using GLMs with model selection by Akaike’s information criterion (AICc).

To distinguish periods of increased time spent per sector from periods of intense travelling, we calculated mean monthly speed anomalies. Monthly time periods were used to be comparable with other *Eudyptes* studies [35, 48] as well as corroborate with the monthly maps produced (see further on). Mean speed per month was calculated by using displacement over time. The anomalies of the monthly speeds from the average across the whole migration period were then calculated, and the two months of the lowest speed anomalies were taken as the “resident” months. The months representative of travelling speeds are called “migratory” months.

### Quantifying spatio-temporal overlaps

To visualise the degree of spatial separation due to allochrony, we simulated a scenario in which allochrony was absent by delaying the departure date of Fiordland penguins by two months to give the same median departure dates as Snares penguins. This method provides a mechanism to disentangle the degree to which allochrony and space use separate the observed migration distributions. Monthly usage maps were produced using the posterior estimates, by summing all possible locations within a month, among all the individuals for each species. This allowed us to produce estimated usage maps incorporating all the information from the estimation process. The greater the usage of an area by multiple animals within a species (*i*.*e*. the number of putative locations in a pixel), the higher the relative importance of that pixel for that species. We plotted the monthly 50% and 90% UDs (using the percentile method described above).

To quantify the degree of spatial separation due to allochrony, we used the weekly overlap statistics to evaluate spatial overlap at a finer scale (than monthly). We used utilisation distribution overlapping statistics for the two scenarios (observed and synchronous) across the number of weeks that spanned the non-breeding periods (30 weeks for the observed migrations and 22 weeks for the synchronous migrations); and calculated the area under the curve (AUC) for each. To make the periods of time comparable we converted the weeks to a percentage of the relative migration time. Utilisation distribution overlapping statistics (as a measure between 0- no overlap- and 1- full overlap: y-axis) were plotted against the relative migration time (x-axis). The differences in the AUC provides an index of the reduction in overlap that arises from allochrony [18]. This was done for both the 50% and 90% UDs.

## Results

Of the 52 devices deployed on Fiordland penguins 28 (54%) were recovered, compared to 34 of the 44 devices (77%) for Snares penguins. Snares penguins breed in large colonies of up to 1,300 nests, whereas Fiordland penguins breed singly or in small groups in dense forest, making recovering devices from birds far more difficult for the latter. The number of devices that had sufficient data to fully estimate locations for the complete non-breeding season was 27 for both Snares and Fiordland penguins (13 for the mainland and 14 for Codfish Island). The poor performance of the Snares tags was due to a manufacturing defect that caused premature battery expiry.

### Overview of spatial use patterns

**Fig 2:**
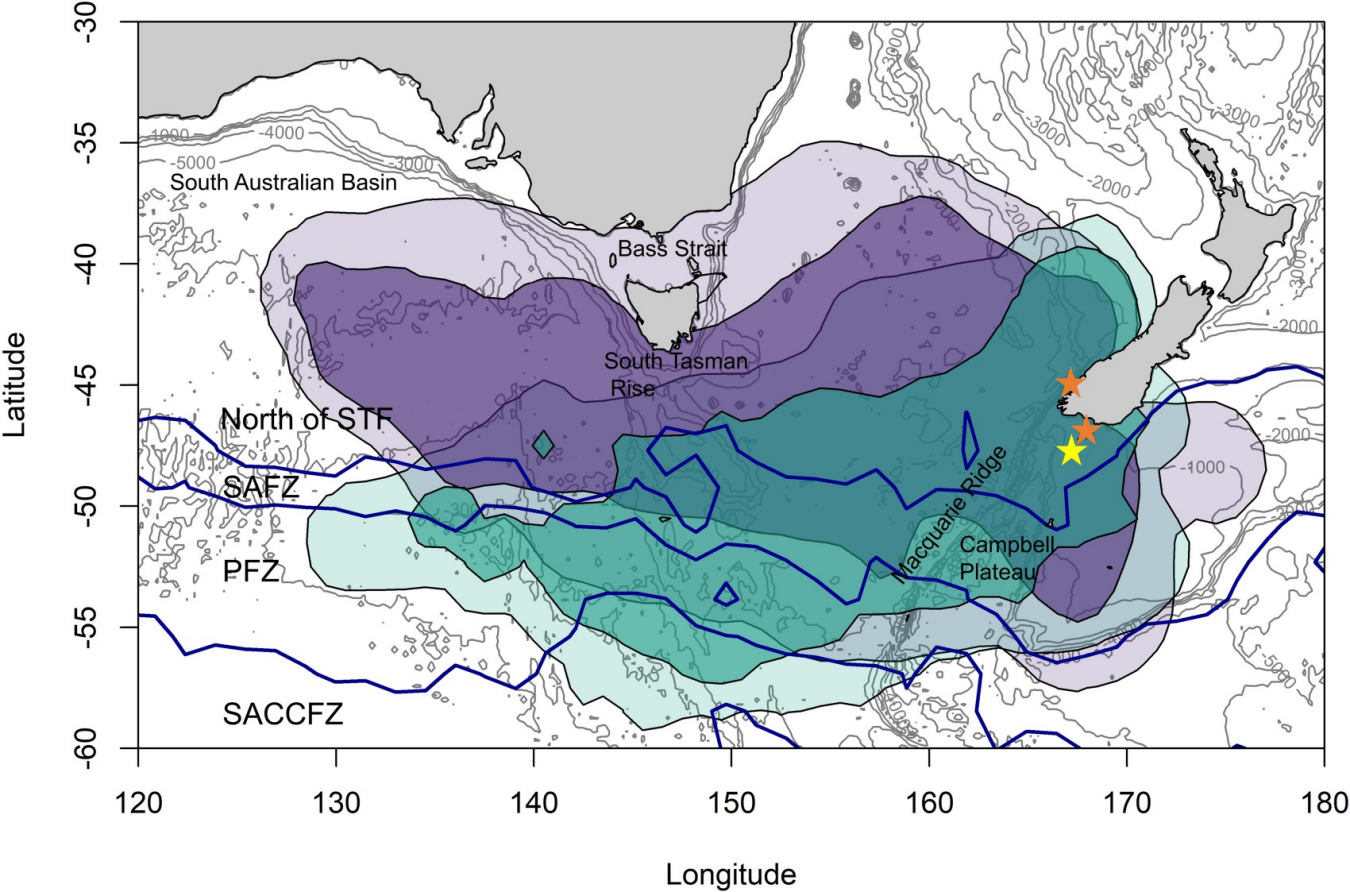
Distribution of Fiordland and Snares penguins during the post-moult non-breeding season. Coloured fill indicates 50% (core usage; darker) and 90% (peripheral distribution; paler) utilisation distributions for Fiordland penguins (turquoise) and Snares penguins (purple). The orange stars indicate the Fiordland breeding colonies along the New Zealand mainland coast of Southwestland and Fiordland and on Codfish Island off the south of New Zealand and the yellow star indicates the Snares Islands where Snares penguins breed. North of STF = North of the Sub-Tropical Front, SAFZ = Sub-Antarctic Frontal Zone, PFZ = Polar Frontal Zone and SACCFZ = Southern Antarctic Circumpolar Current Frontal Zone. Grey lines represent bathymetric contours with 1000 m interval.

Both Snares penguins and Fiordland penguins showed consistent, directed migrations of similar extents to the west of New Zealand in oceanic waters with depths of over 3,500 m (Fig 2). However, Snares movements were oriented more to the WNW, in relation to their colony, such that they remained mostly north of the Sub-Tropical Front (STF). The Fiordland penguins’ outbound migration was orientated to the WSW, in relation to their colonies, crossing three fronts and making use of the Sub-Antarctic Frontal Zone (SAFZ), Polar Frontal Zone (PFZ) and Southern Antarctic Circumpolar Current Frontal Zone (SACCFZ) (Fig 2). They returned to their colonies via a more northerly orientation, using the waters north of the STF for their inbound trip.

**Fig 3:**
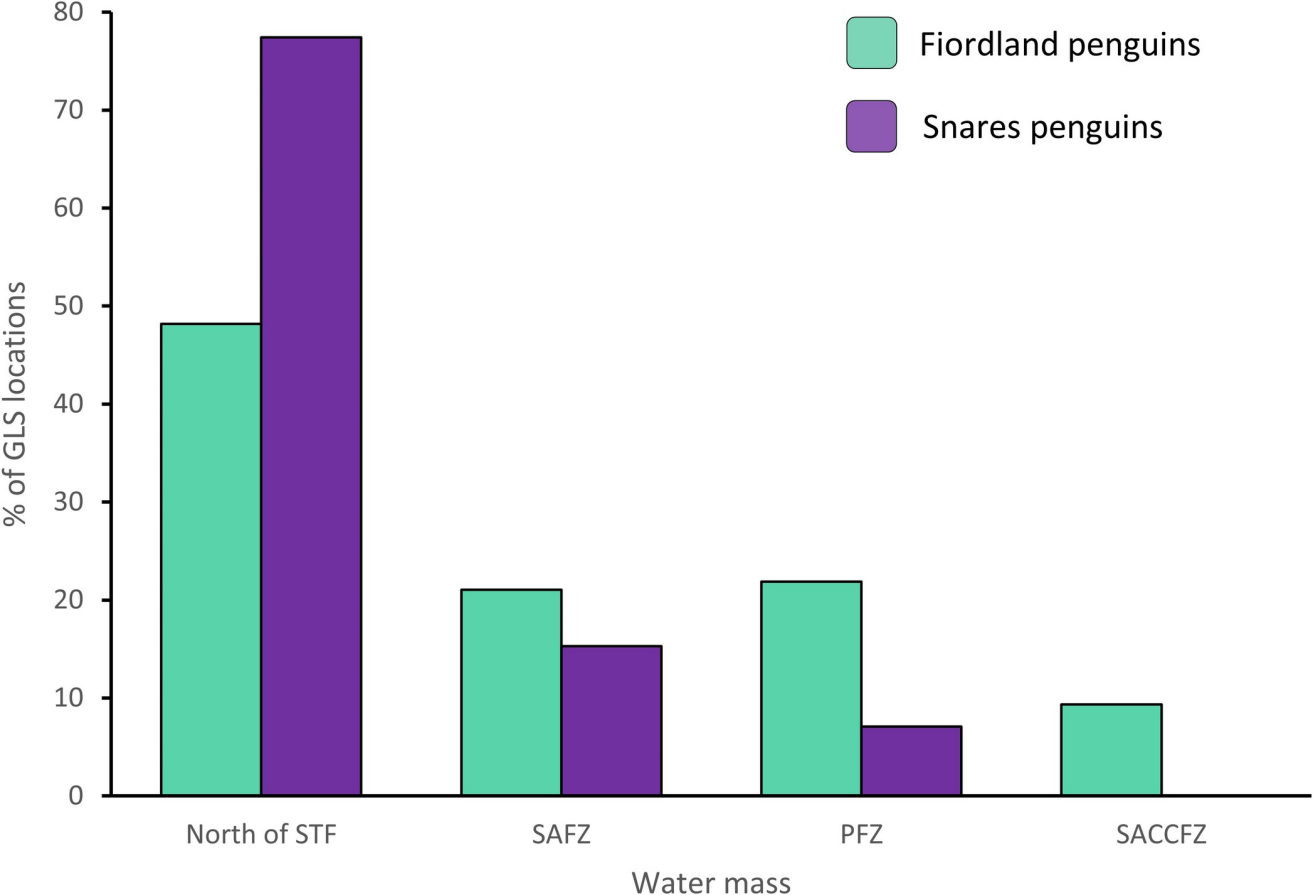
Usage of the different water masses by Fiordland penguins and Snares penguins during the non-breeding season. Water masses are: North of STF = north of the Sub-Tropical Front, SAFZ = Sub-Antarctic Frontal Zone, PFZ = Polar Frontal Zone and SACCFZ = Southern Antarctic Circumpolar Current Frontal Zone.

The water mass north of the Sub-Tropical Front and three inter-frontal zones were used by the two species within the 90% UDs. Snares penguins were located mainly North of the STF (77.5%), returning to their breeding colony via the SAFZ (15.3%) and PFZ (7.01%; Fig 3). The percentage of posterior location estimates for Fiordland penguins located North of the STF was 48.2%; with the other 51.8% of locations in the SAFZ (20.6%) and PFZ (21.9%) and, to a lesser extent, the SACCFZ (9.3%).

### Trip metrics

Both species were at sea for approximately four and a half months (139 ± 11 days and 136 ± 8 days for Fiordland penguins and Snares penguins, respectively). Broadly, trip characteristics were similar in terms of their duration and the cumulative distances travelled, with expected marked differences in the timing of migration (Tables 1 and 2). Unexpectedly, there were differences between the maximum distances swum from the colonies for the two species (Table 2), with Snares penguins travelling slightly further distances than Fiordland penguins (Table 1). Reflecting the typical biology of *Eudyptes* penguins, females from both species returned ashore after males, which is reflected in the females accumulating longer swimming distances over the non-breeding season than males.

**Table 1:** Summary of track statistics (±SD) for Fiordland penguins and Snares penguins tracked during the post-moult dispersal period using light based geolocators.

Species		N	mean departure date	mean return date	days at sea	cumulative distance travelled (km)	max distance from colony (km)
Fiordland	All	27	27 Feb ± 6 days	16 July ± 8 days	139 ± 11	6, 069 ± 969	1, 876 ± 523
	Female	14	26 Feb ± 1 day	22 July ± 6 days	146 ± 7	6, 359 ± 992	1, 903 ± 621
	Male	13	28 Feb ± 1 day	10 July ± 6 days	132 ± 9	5, 756 ± 875	1, 847 ± 412
Snares	All	27	25 April ± 4 days	8 Sept ± 7 days	136 ± 8	6, 443 ± 1, 153	2, 221 ± 599
	Female	14	24 April ± 1 day	10 Sept ± 3 days	139 ± 8	6, 707 ± 1, 003	2, 244 ± 601
	Male	13	26 April ± 1 day	5 Sept ± 3 days	132 ± 6	6, 135 ± 1, 282	2, 193 ± 621

N denotes sample size.

**Table 2:** Backwards elimination step-wise models of differences for trip characteristics between Fiordland penguins and Snares penguins tracked during the post-moult dispersal period.

Model	Term	AIC	ΔAIC
departure date ~ Species*Sex	**departure ~ Species**	**388.64**	**0**
	departure ~ Species: Sex	389.58	0.94
	departure ~ Sex	455.74	67.1
return date ~ Species*Sex	**return ~ Species**	**686.7**	**0**
	return ~ Species: Sex	690.4	3.7
	return ~ Sex	713.83	27.13
days at sea ~ Species*Sex	**days ~ Species: Sex**	**218.5**	**0**
	days ~ Sex	219.23	0.73
	days ~ Species	241.41	22.91
cumulative distance travelled (km) ~ Species*Sex	**dist ~ Sex**	**736.28**	**0**
	dist ~ Species: Sex	738.03	1.75
	dist ~ Species	739.9	3.62
max range from colony (km) ~ Species*Sex	**max range ~ Species**	**669.22**	**0**
	max range ~ Species: Sex	672.89	3.67
	max range ~ Sex	676.07	6.85

Species:Sex in the Term column refers to the interactions between Species and Sex.

**Fig 4:**
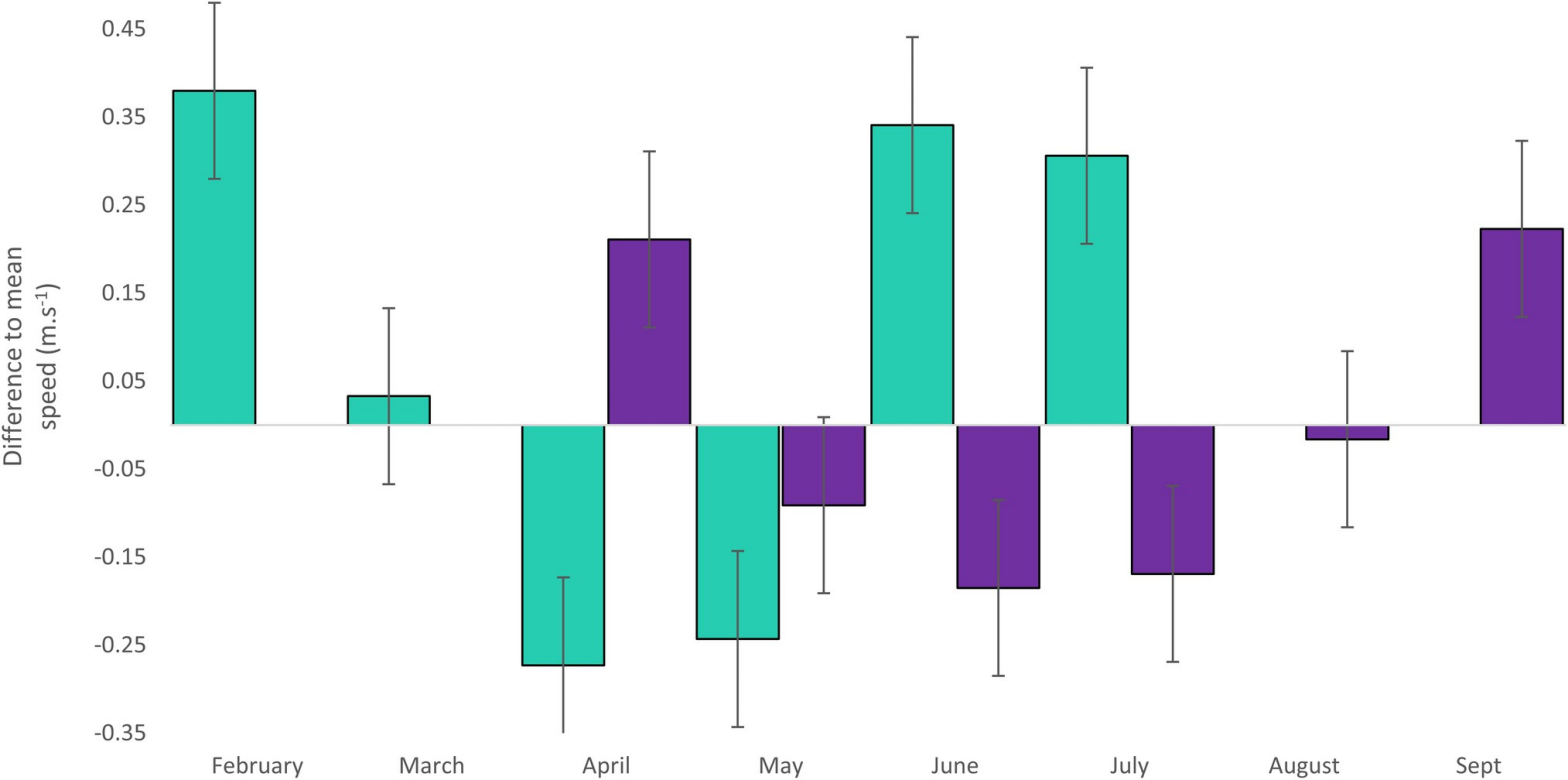
Monthly anomalies from trip speed for tracked Fiordland penguins and Snares penguins over the non-breeding season. The Fiordland penguin migration takes place from February to July, while the Snares penguin migration takes place from April to September. Mean individual travelling speeds were 0.57 ± 0.4 m.s^-1^ and 0.63 ± 0.4 m.s^-1^ for Fiordland penguins and Snares penguins, respectively. Fiordland penguins had the slowest speeds during the months of April and May, and Snares penguins during the months of June and July.

Fiordland penguins had the slowest speeds during the months of April and May, and Snares penguins during the months of June and July (Fig 4). These are considered the resident periods. Overall, mean travel speeds were 0.57 ± 0.4 m.s^-1^ and 0.63 ± 0.4 m.s^-1^ for Fiordland penguins and Snares penguins, respectively while those during the resident period were 0.46 ± 0.1 m.s^-1^ and 0.45 ± 0.2 m.s^-1^, respectively. Speeds were significantly lower during the resident period compared to the migratory period for Fiordland penguins (Student’s t-test, t_16080_ = 19.5, p = .01), but not for Snares penguins (Student’s t-test, t_17304_ = 4.7, p = .09).

### Spatio-temporal overlaps

**Fig 5:**
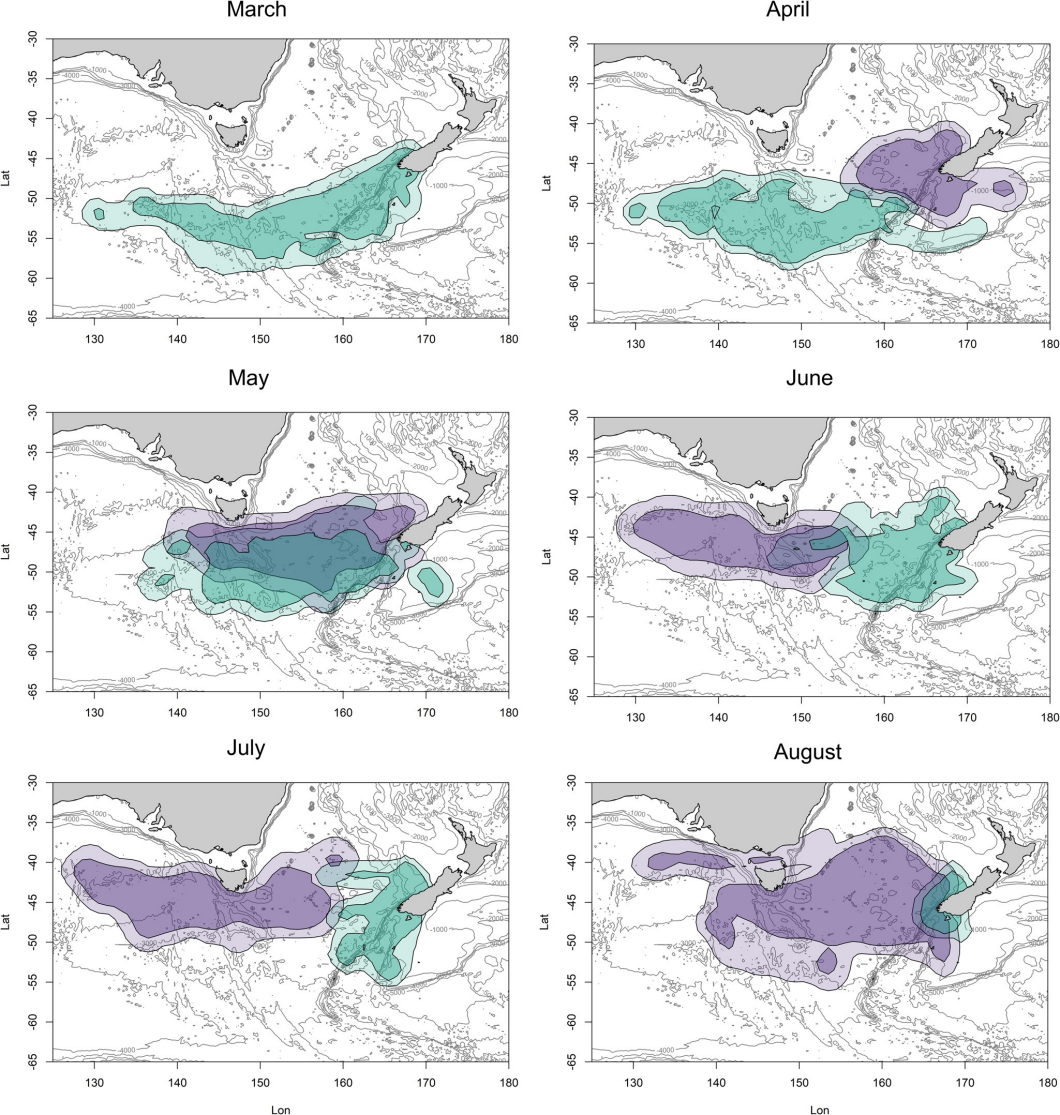
The core (50% UD) and peripheral (90% UD) non-breeding distributions for Snares (purple) and Fiordland (turquoise) penguins. February (the month when the Fiordland penguins first set out) and September (when the Snares penguins return to their colonies) were excluded as they only represented one week of data each.

The Fiordland penguins move rapidly at the start of their migration so that when the Snares penguins set out in April, there is very little overlap in their distributions (Fig 5). There could be some unquantified overlap with Snares penguins on pre-moult trips at the end of February and during March when Fiordland penguins commence migration, but this will decline to zero by April when Snares penguins are moulting ashore. During May, the two species’ paths cross: Fiordland penguins are in one of their resident months and Snares penguins are outbound towards their resident wintering area (Fig 5: May). There is very little or no overlap for the months of June and July when the crossover of the two species’ return and outbound paths are completed. Then, there is again an increase in spatial overlap at the end of August when the Snares penguins are travelling back to their breeding island, congregating with Fiordland penguins that are foraging in the vicinity of their colonies.

**Fig 6:**
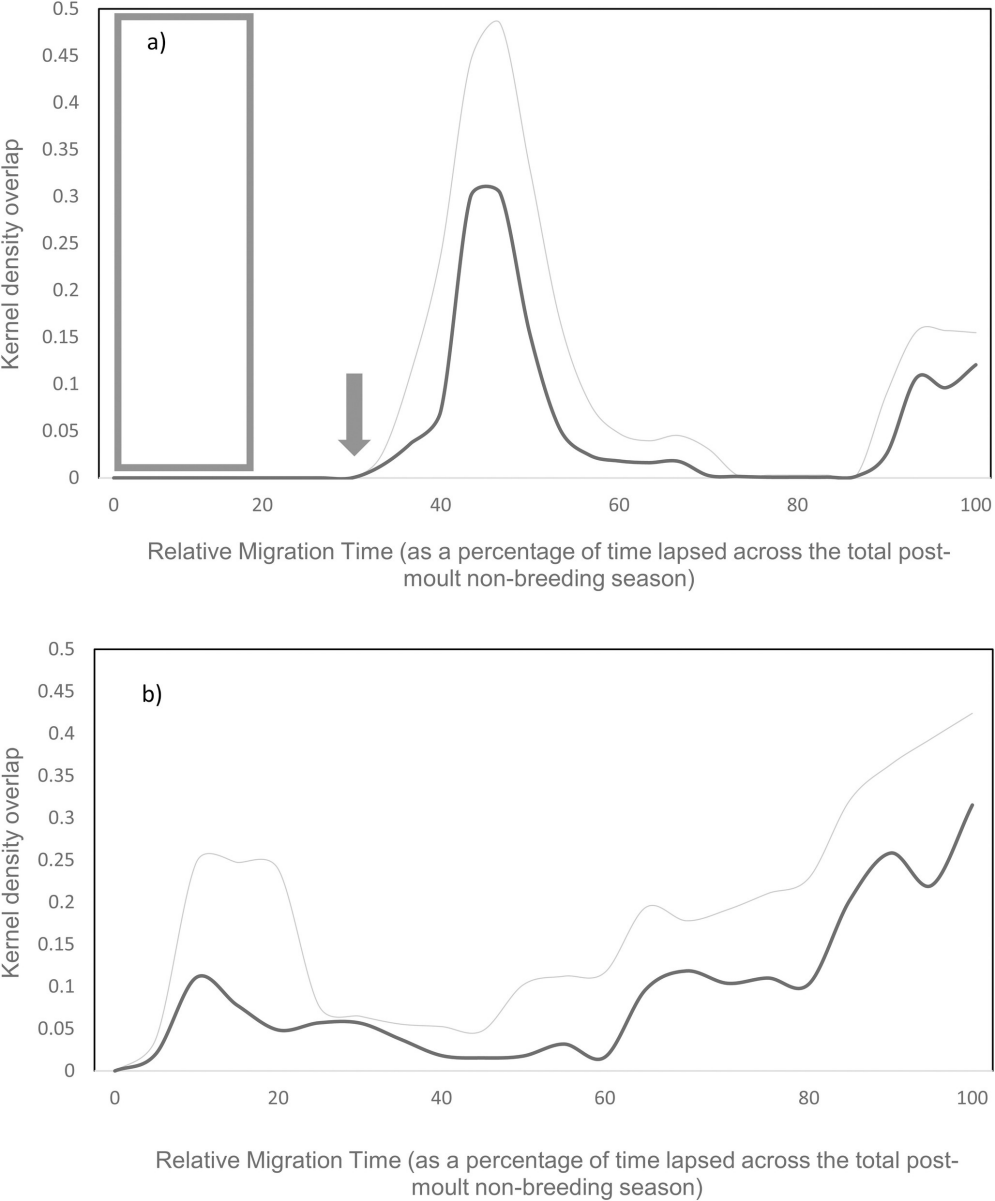
Weekly Utilisation distribution overlapping statistics and area under the curve comparisons between observed and synchronous migrations. Overlap indices for the core area (50% isopleth; dark grey) and the peripheral utilisation distributions (90% isopleth; light grey) for a) the observed and b) the synchronous migrations for Snares and Fiordland penguins during their non-breeding season presented with the relative percentage of time lapsed across the non-breeding season. The period of time in a) runs from the start of the Fiordland penguin migration (at the end of February) to the end of the Snares migration (which ends at the beginning of September). At the start of the observed migrations Fiordland penguins embark on their migrations. After 8 weeks, the Snares first start their migration (indicated by the arrow). The dark grey rectangle (a) represents the period when the Snares penguins would still be on their pre-moult trip (overlapping with the post-moult migration of the Fiordland penguins). During the synchronous migrations (b), Snares and Fiordland penguins were assumed to leave and return simultaneously.

When quantifying the spatial overlap across the migration, there is no overlap for the first 30% of the migration (not accounting for the Snares pre-moult trip; Fig 6A: grey rectangle). There is a steep rise in overlap between the two species between 40–55% into the migration, reaching a peak overlap index of 0.48 (Fig 6A). This coincides with the middle two weeks of May. This peak falls steeply afterwards, maintaining low overlap indices, of less than 0.05 in the peripheral area, from 60 to 87% (with the highest overlap indices being 0.02 in the core area and 0.05 in the peripheral area). This period is during the middle of the Snares non-breeding season and the end of the Fiordland penguin migration. The last 15% of the migration reaches a peak overlap index of 0.16 at the end of August and beginning of September.

**Fig 7:**
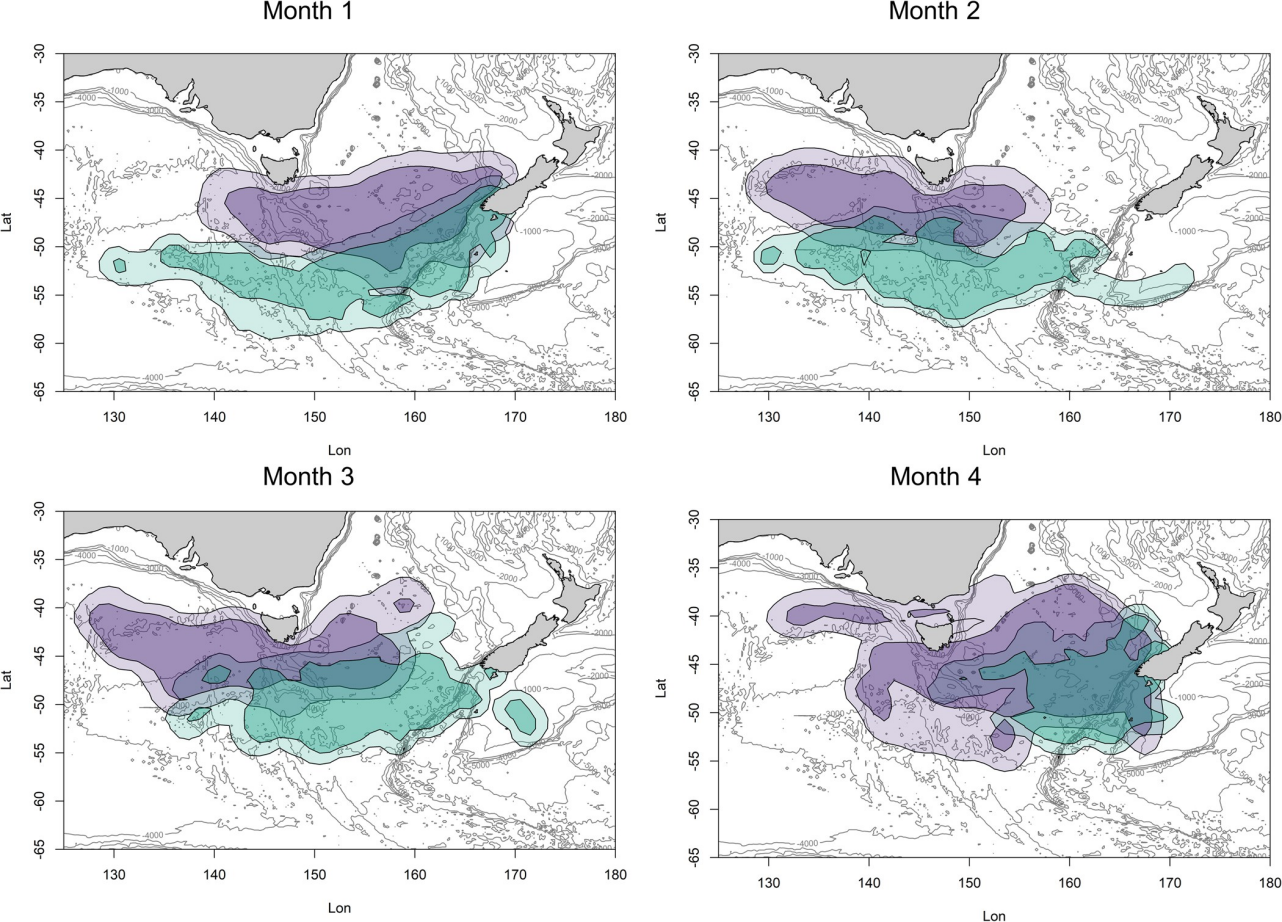
The synchronous core (50% UD) and peripheral (90% UD) non-breeding distributions for Snares (purple) and Fiordland (turquoise) penguins. The start of the Fiordland penguins migration was shifted later in time by two months to coincide with the start of the Snares penguins migration (*i*.*e*. the Fiordland penguin distribution for March was shifted to May, etc.). February (the month when the Fiordland penguins first set out) and September were excluded as they only represented one week of data each.

If the two species migrated in synchrony, spatial overlap would be present throughout the non-breeding season (Figs 6B and 7). When viewed at a weekly timescale, overlap under this scenario occurs more consistently through the season compared to the observed situation with allochrony (Fig 6A vs 6B). At the onset of the synchronous migrations, there would initially be an increase in spatial overlap (Fig 6B: 0–25%) in the area south-west of New Zealand (Fig 7) when both species would be departing their colonies after the moult. As Fiordland penguins have a more southerly distribution, the utilization distributions diverge and the overlap indices decline. This period of relatively low overlap accounts for just 20% of the relative migration period (from 25–45%). From 60% into the migration, the overlap steadily increases to an overlap index of 0.3 (core area) and 0.42 (peripheral area) at the end of the migration, when the birds would all be gathering around their breeding colonies (Figs 6B and 7).

Comparing the AUC values of Fig 6A and 6B quantifies the reduction in overlap in seasonal Snares and Fiordland penguins’ utilisation distributions that arise from allochrony. If the penguins were to start their migrations synchronously, overlap would increase by 36.60% and 29.58% in the core area and peripheral area, respectively, over the entire non-breeding season.

## Discussion

The migrations paths of the Fiordland penguins tracked in this study were similar in orientation and distance to Mattern et al. [39] and Thiebot et al. [38] who tracked Fiordland penguins during the pre-moult trip and post-moult migrations. This provides support for the migration paths of Fiordland penguins being consistent across years, which provides confidence that comparisons of tracks with congeners during different years are valid. These are the first published migration tracks for Snares penguins, providing the opportunity to describe spatio-temporal partitioning between these two closely related species and the factors that may influence this.

Both species showed directed migrations of similar extents westwards of New Zealand, but Snares penguins migrate west and stay north of the STF whilst Fiordland penguins head south-westerly, reaching as far as the SACCFZ and then returning to their colony by crossing back over the STF. Niche conservatism between the breeding and non-breeding seasons is a common strategy for seabirds [49, 50]. Snares penguins exhibit a niche conservatism strategy [18], where animals remain in similar habitats during their non-breeding season to those they utilise during the breeding season, comparable to that of austral winter migrating Macaroni penguins at Kerguelen [50]. In comparison, Fiordland penguins adopt a hybrid strategy involving niche switching [18] by foraging in water masses at higher latitude, than those surrounding their breeding colonies, during their outbound migration and resident period, then a niche conservatism strategy on the return journey to their colony across the STF.

Allochrony usually evolves between species that breed in sympatry and show high character displacement, as in the case of MacGillivray’s prions (*Pachyptila macgillivrayi*) and broad-billed prions (*P*. *vittata*) from Gough Island that show a high spatial overlap between migration distributions in the absence of allochrony [14]. In contrast, our findings are more similar to those of Quillfeldt et al. [22] who found contrasting migration strategies between three species of sympatric *Procellariidae* that also exhibit both allochrony and low spatial overlap in non-breeding distributions. Quillfeldt et al. [22] hypothesize that competition may not be the selective pressure that resulted in the phenology and migration strategies differing between sympatric and closely related species that show contrasting migration strategies, but rather these aspects evolved to exploit different peaks in food availability. By attempting to separate the spatial and temporal components of segregation, we aimed to ascertain the mechanisms behind niche differentiation between the Snares and Fiordland penguins. When hypothetically assuming the two species migrate synchronously, overlaps increase, but not to high levels as the niche switching strategy of Fiordland penguins lead their utilisation distributions to be further south during the outbound and resident phases. Segregation then arises from the species using different areas as well as using the same areas at different times which may suggest, similar to Quillfeldt et al. [22] findings, there are other factors than inter-specific competition playing a role in coexistence [51] between these two species.

### Drivers and consequences of niche differentiation

The conflict between resource demand and availability in the winter has lead to different strategies between seabirds that over-winter in high latitudes to meet the energy demands of survival [14, 52]. More specifically diving seabirds, as visual predators, need to balance high energy requirements needed for thermoregulation and challenging conditions of seasonal lows in productivity with reduced day light hours to forage at depth in the winter months [53, 54]. The contrasting foraging areas and times of migration of the two penguin species in this study have allowed these species to effectively bypass the worst of the challenges of foraging during winter in the higher latitudes of the Southern Ocean. The earlier migration of Fiordland penguins allows them to exploit the higher productivity and longer day lengths that occur further south in the SAFZ and PFZ during the austral autumn months compared to the austral winter. Indeed, over a similar period in the late summer and early autumn months, these colder waters in the SAFZ/PFZ support large biomasses of marine predators, including other *Eudyptes* species which would be on their pre-moult trip [29, 55, 56]. Thereafter, with the seasonal change and onset of winter, when the mixed layer depth in the SAFZ/PFZ increases to depths exceeding 500m [57], Fiordland penguins move north and eastwards, and Snares penguins set out on their migration, with most individuals foraging in the waters north of the STF where mixed layer depth is substantially shallower and day length is longer.

These same movements used to take advantage of the productive cold waters further south during the autumn months are reflected in the movements of other Sub-Tropical Frontal Zone breeding birds. Northern Rockhopper penguins from Amsterdam Island in the Southern Indian Ocean move south into higher latitudes during their non-breeding season occupying the same non-breeding area over the austral autumn as allopatric breeding Eastern Rockhopper penguins from Kerguelen do over the austral winter months [23]. MacGillivray’s prions from Gough Island are also austral autumn migraters which have the majority of their non-breeding distribution south of the Sub-Tropical Front [14]. It may be that there is a physiological cost to a Sub-Tropical Frontal Zone breeding species in foraging further south [58]. This may explain why it is beneficial for Fiordland penguins to forage earlier in the year and for Snares penguins to mainly stay north of the STF over the winter months. We hypothesize that were Snares and Fiordland penguins to migrate synchronously, the increase in overlap that we estimated would be under-represented as it is likely then that the delay in migration would make conditions further south unsuitable for Fiordland penguins, resulting in them remaining north of the STF, as the Snares penguins do, and a higher spatial overlap in resource use. This is further corroborated by the fact that most of the Fiordland penguins used the water mass north of the STF when the winter season started.

Snares and Fiordland penguins are likely using different frontal zones to exploit different prey communities. During the breeding season Fiordland penguins feed primarily on fish and Arrow squid (*Nototodarus sloani*) [59] and Snares feed primarily on euphausiid *Nyctiphanes australis* [60] that are coastal species unavailable across their observed winter distributions. Stable isotope studies of the winter diets of *Eudyptes* penguins elsewhere show that pelagic zooplankton account for >84% of their diets [49]. Composition of large zooplankton communities in the Southern Ocean differ among the frontal zones [61]. The SAFZ/PFZ used by Fiordland penguins during their outbound migration and resident period is dominated by Sub-Antarctic species such as *Euphausia lucens*, *E*. *vallentini*, as well as *Thysanoessa gregaria*, *Primno macropa*, *Themisto gaudichaudii* [61], the same species found in the diets of Eastern rockhoppers and Macaroni penguins that forage in these frontal zones [62]. Communities north of the STF are dominated by *E*. *similis var*. *armata*, *E*. *spinifera* and *Thysanoessa gregaria* [61, 63] which are likely to form the diets of Snares throughout their non-breeding period, and Fiordland penguins during their return migration.

### Explanations for allochrony

Penguins experience two periods of hyperphagia (or persistent feeding): before the catastrophic moult (when penguins moult all their feathers simultaneously) and before the breeding season starts. Penguins fast for three to four weeks ashore throughout the moult [33]. At the start of the breeding season, penguins again need to fast for up to 40 days during the courtship and incubation periods. During these periods of hyperphagia, penguins are particularly susceptible to intra- and interspecific competition for prey resources. Macaroni penguins have been shown to experience the highest pressure to forage effectively over the annual cycle immediately before returning to land to breed [37]. Although not a *Spheniscidae*, this has also been found true for the Common guillemot (*Uria aalge*) as well, a seabird with high wing-loading and similar pursuit diving to penguins [54]. This reveals that contrary to what most researchers assume, the greatest energy requirements for some seabird species does not take place during the breeding season, but instead at the end of the non-breeding migration in preparation for the breeding attempt. Alternatively, birds simply forage more during this time as it may be that there is decreased prey availability in the area between the resident non-breeding foraging area and their colonies [37]. Regardless, prior to their return to the colony, the penguins must be feeding intensively to maintain good body condition to survive the fasting period [34]. Our findings show that during synchronous migrations, Snares and Fiordland penguins would experience a substantial increase in overlap at the end of the non-breeding season both in duration and in space when high number of birds would be converging at relatively high density close to the colonies. However, while allochrony has resulted in there being no spatial overlap around the colonies for returning Fiordland penguins, there is an increase in spatial overlap when Snares penguins are returning to their colonies. The timing of Snares penguins returning coincides with Fiordland penguin chick hatching and female Fiordland penguins foraging in the vicinity of their colonies. Had competition for resources been the mechanism for allochrony, we would expect that the life cycles would be offset by a few weeks, as with other sympatric *Eudyptes* penguins, and not two months, as only one species secures the benefit of arriving in the absence of the other.

An alternative explanation for allochrony arising due to competition [14], is that allochrony may arise among congeneric seabirds due to differences in ocean regimes between their colonies that can drive speciation through parapatry or allopatry [64]. Differences north and south of the Subtropical Convergence drove the speciation of Rockhopper penguins (*Eudyptes chrysocome*) breeding in these two areas into the Southern and Northern rockhopper species [65] roughly 0.9 million years ago [66]. Summer breeding is the dominant strategy employed by *Eudyptes* penguins and so, to reduce competition between closely related competitors, we could expect a shift in phenology which could result in a winter breeding niche. Southern and Northern rockhopper penguins, like Snares and Fiordland penguins, have distinct mitochondrial DNA and also show marked allochrony. However, Northern rockhopper penguins have evolved to breed in late winter/early spring, 8 weeks before Southern rockhoppers in the absence of competition from other diving seabird species [67]. Comparably, differences in ocean regime based on local productivity around breeding colonies may have resulted in the speciation between Snares and Fiordland penguins which diverged from their common ancestor around 0.5–1.4 million years ago [32].

There is considerable evidence that seabird behaviour and performance are driven largely by food availability [68]. As Snares and Fiordland penguins breed in parapatry and not sympatry, it is more likely that niche divergence results from the difference in the locally abundant prey resources and suitable environmental conditions around their colonies. This explains why the optimal timing for breeding differs between the two species despite the proximity of their colonies. The shift in phenology of Fiordland penguins was made possible by the locally abundant spawning Arrow squid and fish prey resources around the west coast of the South Island at the end of winter/beginning of spring [34, 69]. When the squid availability decreases in the summer months [70], Fiordland penguins enter their non-breeding season. They can then forage in more distant oceanic frontal zones [38, 39] allowing them to track abundant prey throughout the year by then taking advantage of the tail-end of the summer’s productivity in the SAFZ/PFZ. The increased productivity of the warm Sub-Tropical waters in the spring and summer months result in the spring bloom of euphausiids as well as fish and cephalopods within range of the Snares Islands [71, 72]. The beginning of the breeding season for offshore foraging Snares penguins is timed to coincide with this bloom.

Reproductive isolation would have evolved between the penguins breeding on Snares Island, south of New Zealand, and the New Zealand mainland coast of Southwestland via philopatry and allochrony as the birds shifted their phenology to match different timing of local prey availability. Due to this, it is less likely that Fiordland penguins adopted earlier breeding because of interspecific competition with Snares penguins driving temporal niche partitioning. It may simply be that foraging and/or breeding conditions are better for them in early spring [70], and allochrony arose from different timings in optimal conditions for breeding at mainland New Zealand [69] and the Snares Islands [60].

### Conclusion

Niche switching has been hypothesised to be a derived trait compared to niche conservatism which is considered the primitive state [22] in the evolution of migration patterns. This suggests migrations north of the STF during the non-breeding season is the original migration strategy, and the foraging ecology of the Fiordland penguins in space and time reflects the plasticity and adaptation to seasonal availability of resources of this species. We found that allochrony underpins segregation of Snares and Fiordland penguins. The earlier departure of Fiordland penguins results in spatial partitioning due to them switching to higher latitude niches, and later in the non-breeding season, allochrony results in temporal partitioning north of the STF when Fiordland penguins and Snares penguins would otherwise congregate around their colonies. This allochrony is likely due to their phenology being matched to peaks in prey availability in their breeding seasons, and any reduction in competition that arises to this is an incidental outcome.

## Supporting information

S1 Fig A comparison between the oceanic fronts for 2013 (blue solid line) and 2018 (black dashed line). Front locations were broadly similar across years.
(DOCX)

S2 Fig Mean coordinates of non-breeding tracks for geolocation tagged penguins. a) Fiordland penguins and b) Snares penguins tracked over the non-breeding migrations from February 2018 to July 2018 and April 2013 to September 2013 respectively. Within a species, most birds dispersed in the same direction, except for one female Fiordland penguin from Codfish Island/Whenua Hou, which spent the entire winter period on the Campbell Plateau, and one Snares penguin which returned via the Bass Strait.
(DOCX)

S3 Fig Standard deviations for the number of posterior distribution locations per pixel from the *SGAT* package for geolocation tracked penguins. a) Fiordland penguins and b) Snares penguins tracked over the non-breeding migrations from February 2018 to July 2018 and April 2013 to September 2013, respectively. The areas of green denote grid cells of high usage by the penguins.
(DOCX)

S4 Fig Distribution of Fiordland penguins from along the New Zealand mainland coast of Southwestland and Codfish Island during the post-moult non-breeding season. The Southwestland Fiordland penguins are represented by the blue 50% (darker polygon) and 90% (lighter polygon) utilisation distributions (UDs). The Fiordland penguins that bred on Codfish Island are represented by the green 50% (darker polygon) and 90% (lighter polygon) UDs. The third map shows that the UDs are sufficiently similar for the data to be pooled for the purposes of this study. This data was collected February 2018 to July 2018.
(DOCX)
