# Supplementary material for: The role of allochrony in influencing interspecific differences in foraging distribution during the non-breeding season between two congeneric crested penguin species
Source: PLoS One. 2022 Feb 9;17(2):e0262901. doi: 10.1371/journal.pone.0262901 (PMC8827451; doi:10.1371/journal.pone.0262901)
Supplement: S1 Fig — Front locations were broadly similar across years. (DOCX) [file pone.0262901.s001.docx]

**
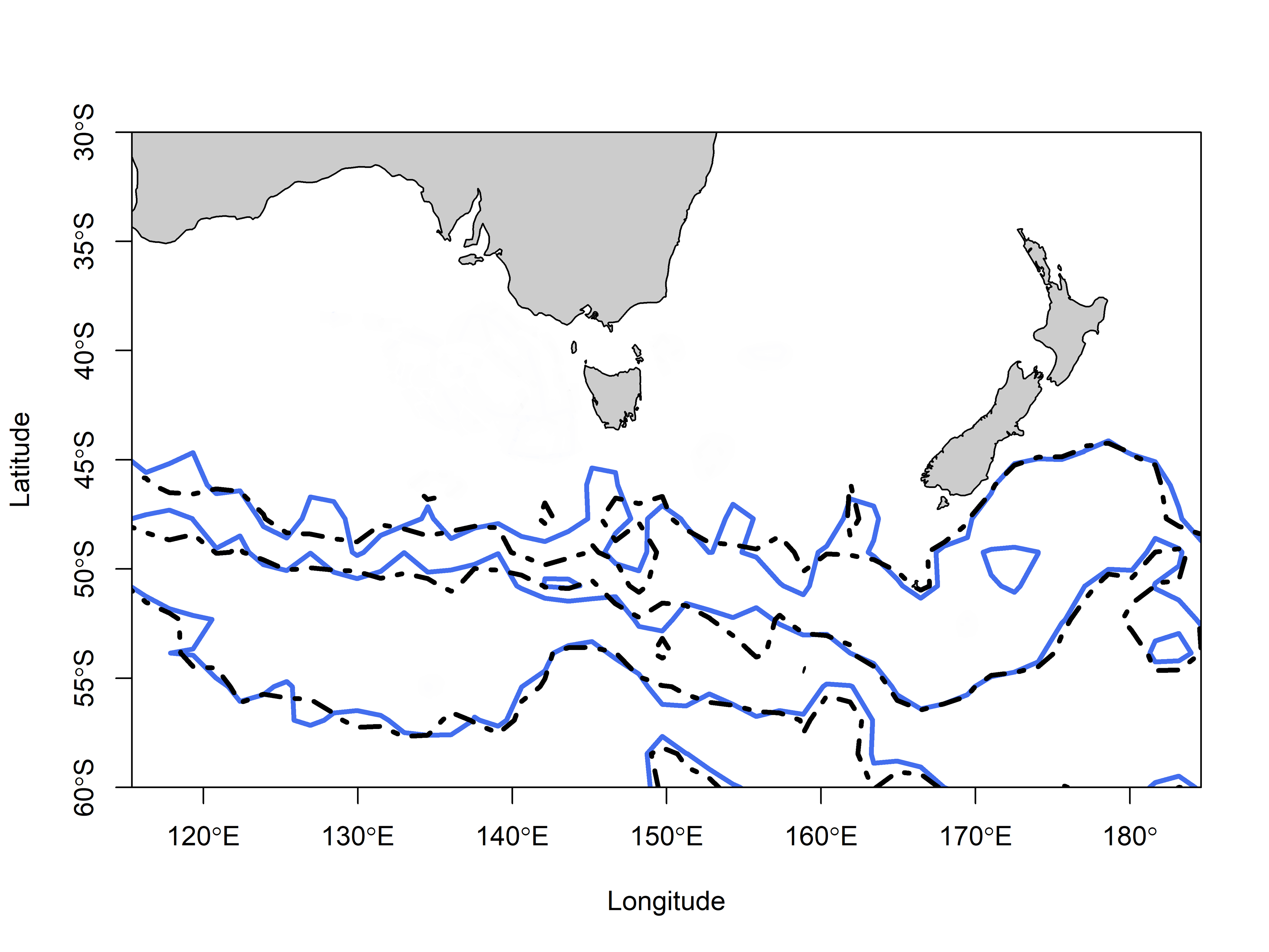
**

**S1 Fig.**  **A comparison between the oceanic fronts for 2013 (blue solid line) and 2018 (black dashed line).** Front locations were broadly similar across years.
