# Supplementary material for: The role of allochrony in influencing interspecific differences in foraging distribution during the non-breeding season between two congeneric crested penguin species
Source: PLoS One. 2022 Feb 9;17(2):e0262901. doi: 10.1371/journal.pone.0262901 (PMC8827451; doi:10.1371/journal.pone.0262901)
Supplement: S2 Fig — a) Fiordland penguins and b) Snares penguins tracked over the non-breeding migrations from February 2018 to July 2018 and April 2013 to September 2013 respectively. Within a species, most birds dispersed in the same direction, except for one female Fiordland penguin from Codfish Island/Whenua Hou, which spent the entire winter period on the Campbell Plateau, and one Snares penguin which returned via the Bass Strait. (DOCX) [file pone.0262901.s002.docx]

**
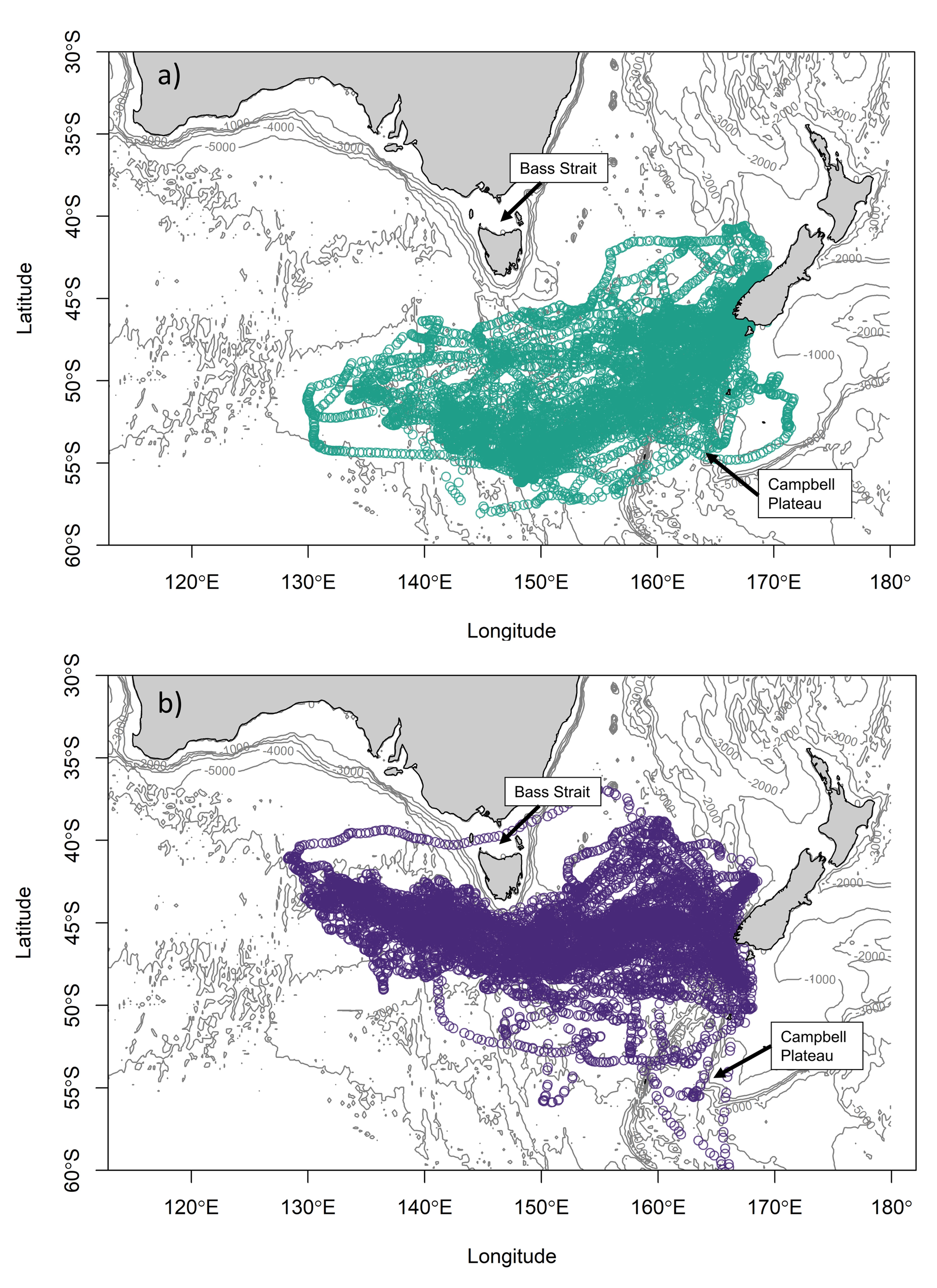
S2 Fig.** **Mean coordinates of non-breeding tracks for geolocation tagged penguins.** a) Fiordland penguins and b) Snares penguins tracked over the non-breeding migrations from February 2018 to July 2018 and April 2013 to September 2013 respectively. Within a species, most birds dispersed in the same direction, except for one female Fiordland penguin from Codfish Island/Whenua Hou, which spent the entire winter period on the Campbell Plateau, and one Snares penguin which returned via the Bass Strait.
