# Supplementary material for: The role of allochrony in influencing interspecific differences in foraging distribution during the non-breeding season between two congeneric crested penguin species
Source: PLoS One. 2022 Feb 9;17(2):e0262901. doi: 10.1371/journal.pone.0262901 (PMC8827451; doi:10.1371/journal.pone.0262901)
Supplement: S3 Fig — a) Fiordland penguins and b) Snares penguins tracked over the non-breeding migrations from February 2018 to July 2018 and April 2013 to September 2013, respectively. The areas of green denote grid cells of high usage by the penguins. (DOCX) [file pone.0262901.s003.docx]

**
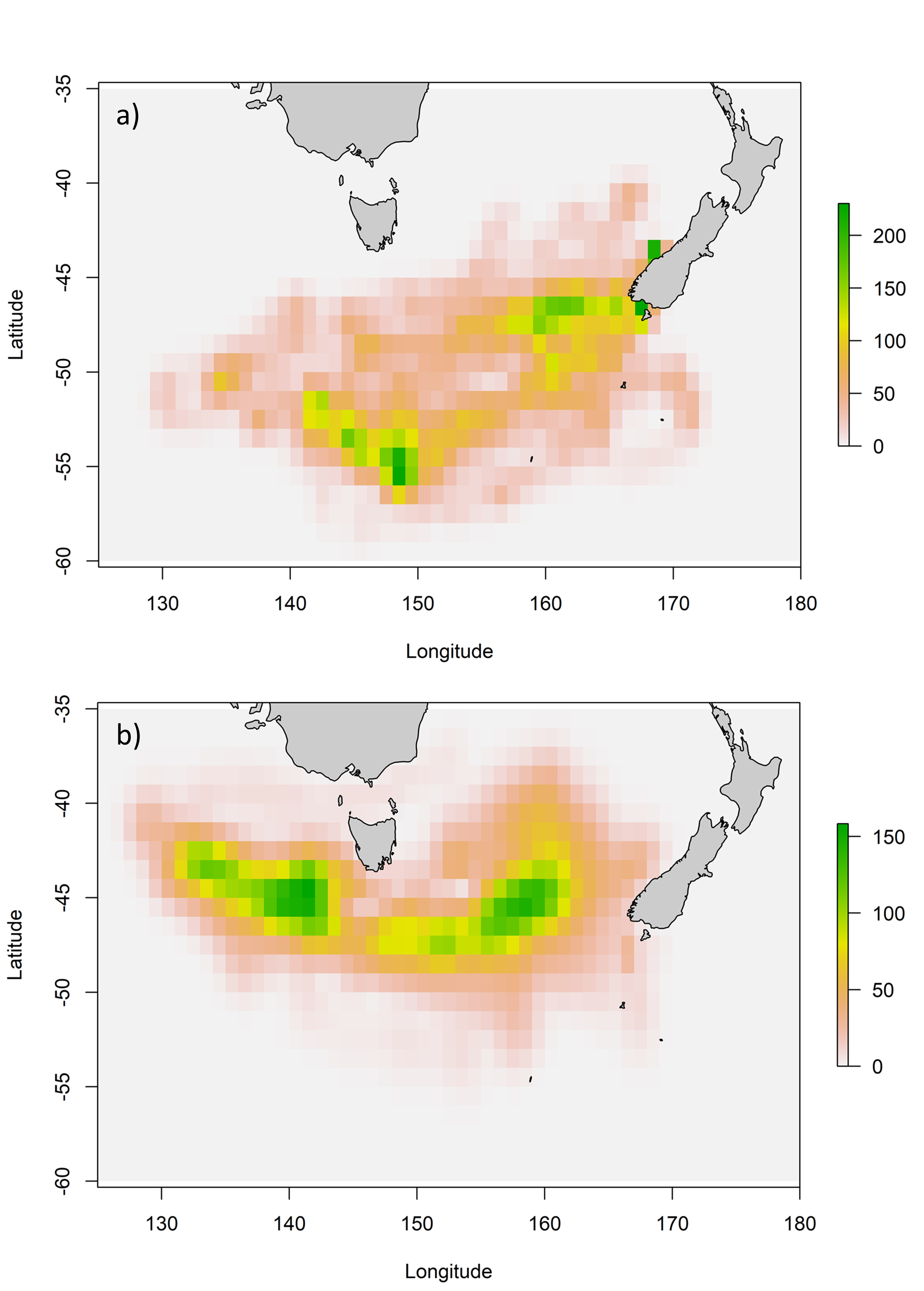
S3 Fig.** **Standard deviations for the number of posterior distribution locations per pixel from the *SGAT* package for geolocation tracked penguins.** a) Fiordland penguins and b) Snares penguins tracked over the non-breeding migrations from February 2018 to July 2018 and April 2013 to September 2013, respectively. The areas of green denote grid cells of high usage by the penguins.
