# Supplementary material for: The role of allochrony in influencing interspecific differences in foraging distribution during the non-breeding season between two congeneric crested penguin species
Source: PLoS One. 2022 Feb 9;17(2):e0262901. doi: 10.1371/journal.pone.0262901 (PMC8827451; doi:10.1371/journal.pone.0262901)
Supplement: S4 Fig — The Southwestland Fiordland penguins are represented by the blue 50% (darker polygon) and 90% (lighter polygon) utilisation distributions (UDs). The Fiordland penguins that bred on Codfish Island are represented by the green 50% (darker polygon) and 90% (lighter polygon) UDs. The third map shows that the UDs are sufficiently similar for the data to be pooled for the purposes of this study. This data was collected February 2018 to July 2018. (DOCX) [file pone.0262901.s004.docx]

**
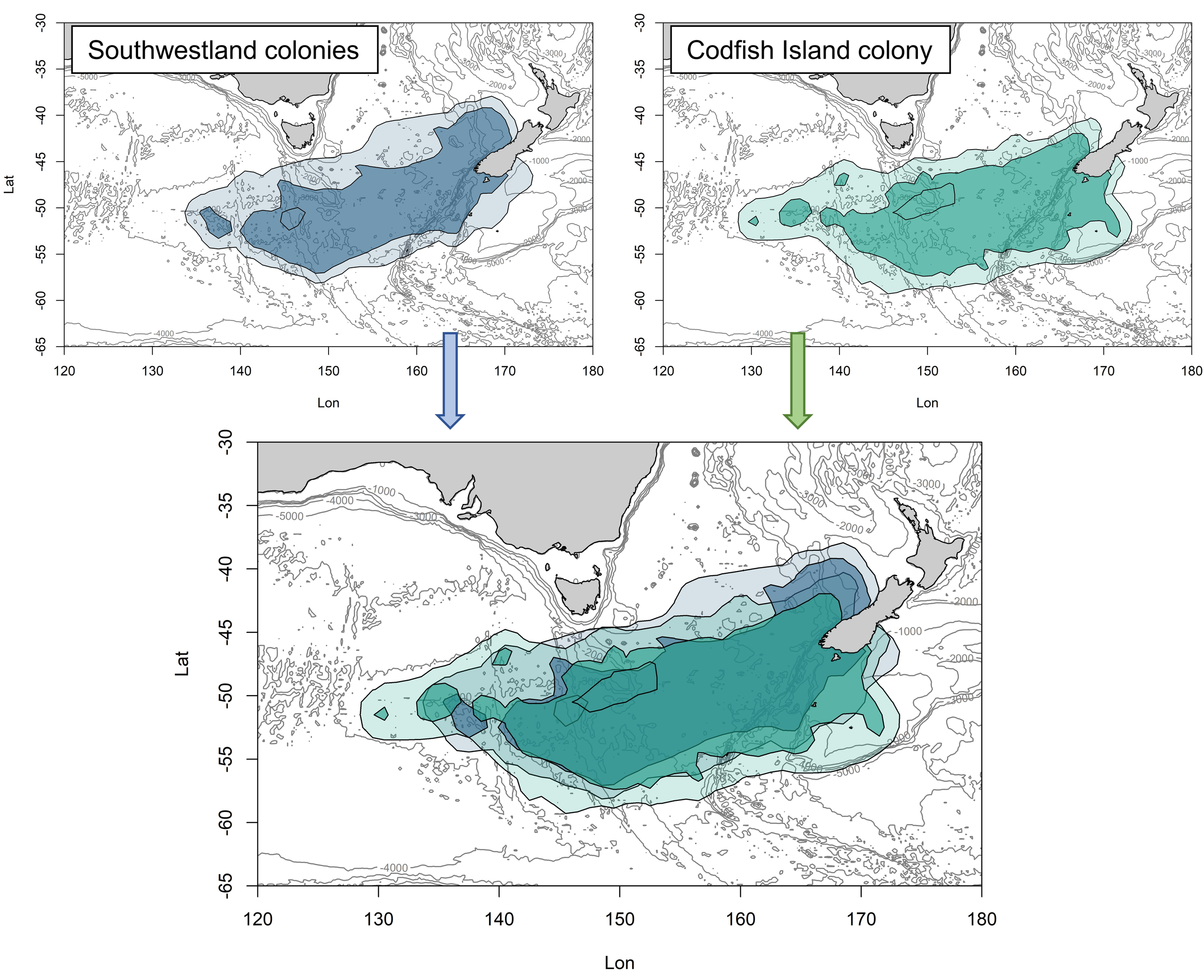
**

**S4 Fig.** **Distribution of Fiordland penguins from along the New Zealand mainland coast of Southwestland and Codfish Island during the post-moult non-breeding season.** The Southwestland Fiordland penguins are represented by the blue 50% (darker polygon) and 90% (lighter polygon) utilisation distributions (UDs). The Fiordland penguins that bred on Codfish Island are represented by the green 50% (darker polygon) and 90% (lighter polygon) UDs. The third map shows that the UDs are sufficiently similar for the data to be pooled for the purposes of this study. This data was collected February 2018 to July 2018.
